# The BRCA1 Breast Cancer Suppressor: Regulation of Transport, Dynamics, and Function at Multiple Subcellular Locations

**DOI:** 10.6064/2012/796808

**Published:** 2012-10-18

**Authors:** Beric R. Henderson

**Affiliations:** Westmead Institute for Cancer Research, Westmead Millennium Institute at Westmead Hospital, University of Sydney, Darcy Road, P.O. Box 412, Westmead, NSW 2145, Australia

## Abstract

Inherited mutations in the *BRCA1* gene predispose to a higher risk of breast/ovarian cancer. The BRCA1 tumor suppressor is a 1863 amino acid protein with multiple protein interaction domains that facilitate its roles in regulating DNA repair and maintenance, cell cycle progression, transcription, and cell survival/apoptosis. BRCA1 was first identified as a nuclear phosphoprotein, but has since been shown to contain different transport sequences including nuclear export and nuclear localization signals that enable it to shuttle between specific sites within the nucleus and cytoplasm, including DNA repair foci, centrosomes, and mitochondria. BRCA1 nuclear transport and ubiquitin E3 ligase enzymatic activity are tightly regulated by the BRCA1 dimeric binding partner BARD1 and further modulated by cancer mutations and diverse signaling pathways. This paper will focus on the transport, dynamics, and multiple intracellular destinations of BRCA1 with emphasis on how regulation of these events has impact on, and determines, a broad range of important cellular functions.

## 1. Introduction

**Figure 1:**
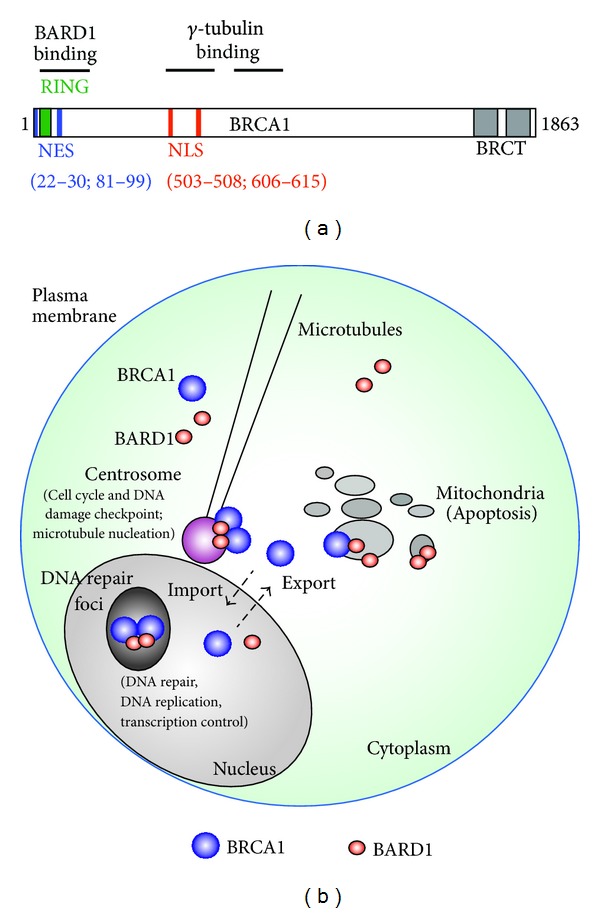
*BRCA1 domain structure and subcellular transport pathways.* (a) Protein domain structure of BRCA1 showing the location of nuclear localization signals (NLSs), nuclear export signals (NESs), and binding sites for BARD1 and gamma-tubulin. The RING and BRCT protein interaction domains are shown at the amino and carboxy termini, respectively. (b) Diagram summarizing the distribution and movement of BRCA1 in the cell. Once translated in the cytoplasm, BRCA1 can move to the centrosome where it dimerizes with BARD1 to ubiquitinate proteins such as gamma-tubulin that regulate centrosome duplication and microtubule nucleation, or to the mitochondria where it is implicated in cell survival and/or apoptosis regulation. BRCA1 enters the nucleus through the importin-alpha/beta pathway and locates at nuclear DNA replication sites. In response to DNA damage, BRCA1 is sequestered (as a dimer with BARD1) to different types of DNA repair complexes at foci. The interaction with BARD1 tends to trap BRCA1 in the nucleus through the masking of its NES. If BRCA1 is not bound to BARD1, it is exported to the cytoplasm by the CRM1 export receptor, and this has been linked to the roles of BRCA1 in apoptosis and in centrosome duplication as revealed by functional comparison of wild-type and NES-mutated forms of BRCA1.

The BRCA1 protein is classified as a tumor suppressor [1]; in healthy cells it functions to maintain proper genomic repair and cell division, but inherited mutations in the *BRCA1* gene encode altered forms of the protein that contribute to development of breast and ovarian cancer [2–4]. Misregulation and reduced expression of BRCA1 also contribute to sporadic forms of breast cancer [5]. The primary tumor suppressing role of BRCA1 relates to the maintenance of genomic integrity through regulation of DNA replication, repair, and transcription, in addition to various cell cycle checkpoints that ensure survival of healthy cells [6]. *BRCA1* gene mutations disrupt these processes and result in chromosome instability and defective checkpoints that accelerate cellular transformation [6–8]. BRCA1 is a multifunctional protein that binds dozens of other proteins, the most important of which is BARD1 [9–11] (see Figure 1(a)). BARD1 forms a stable heterodimer with BRCA1, stimulating its nuclear localization and ubiquitin E3 ligase activity. While the *BARD1* gene, also regarded as a tumor suppressor, is susceptible to germ-line and somatic mutations, these occur at a much lower frequency in a subset of breast/ovarian cancers [12–14]. 

 In recent years it has become accepted that the major cell regulatory proteins (e.g., tumor suppressors) perform many different roles throughout the cell. BRCA1 is a prime example of such a protein: it is actively imported into the nucleus to regulate DNA replication and stimulate gene transactivation, redistributes in the nucleus to sites of damaged DNA to facilitate repair, and is exported to the cytoplasm where it is recruited to centrosomes, to maintain optimal mitotic cell division and cytoskeletal shape, and to mitochondria where it has predicted roles in apoptosis and mitochondrial genome repair (Figure 1(b)). The ability of BRCA1 to shuttle between such diverse locations within the cell, where it forms distinct protein complexes with different protective roles, is a highly regulated and complex process. Previous reviews have touched on the basic pathways of BRCA1 nuclear transport [15–17], which my team has helped define. However during the past few years a wave of new insights into the regulation of BRCA1 transport and dynamics at a wide range of cellular sites has arisen, and this paper will primarily focus on this topic. The reader is directed to other excellent reviews that provide more in depth overviews on the role of BRCA1 in DNA repair and DNA damage response [8, 18–22], transcription function [23, 24], clinical manifestations and correlations [7, 25, 26], and other structure-function aspects of BRCA1 [24, 27]. In this paper, I will refer to these functional and cancer-related aspects of BRCA1 in the context of how they are linked to, and regulated by, the dynamic trafficking of BRCA1 throughout the cell. 

## 2. Subcellular Localization 

### 2.1. The Nucleus

**Figure 2:**
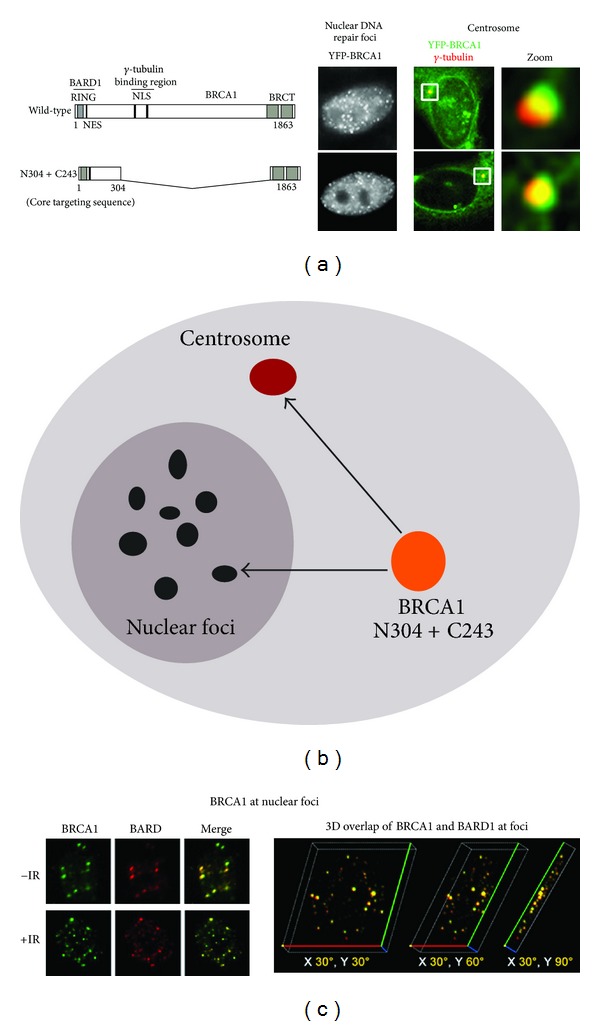
*BRCA1 targeting to centrosomes and nuclear foci.* (a) Deletion mapping studies identified the same minimal targeting sequences (the amino and carboxy termini combined) that were required and sufficient to target BRCA1 to nuclear foci [45] and the centrosome [46]. (b) This is illustrated by a summary diagram. (c) BRCA1 is localized to nuclear foci when analysed by immunofluorescence microscopy. The BRCA1 staining correlates with large visible nuclear spots thought to be DNA replication sites in untreated S-phase cells (see -IR cell images), and redistributes to smaller DNA repair foci after 3 h treatment with 10 Gy ionising radiation (+IR). The images shown are of a single cell nucleus and reveal the typical costaining observed between BRCA1 (green) and BARD1 (red), with overlap displayed as yellow (left-hand image). The overlap in staining is also visible in a three-dimensional rotation of the images (right-hand image; adapted from [47]).

Since its discovery and cloning in 1994 [2], there has been a slow stepwise progression in our understanding of the subcellular distribution of BRCA1, hampered often by technical problems attributable to cross-reactivity and low specificity of certain BRCA1 antibodies. During the 1990s, BRCA1 was detected in the nucleus [28–30], at cytoplasmic granin-associated membranes [31], cytoplasmic tube-like invaginations in the nucleus [32], and in the cytoplasm [29, 33]. In recent years, BRCA1 cellular localization has been studied by combinations of microscopy and cell fractionation/western blotting approaches, enabling us to conclude that BRCA1 becomes phosphorylated and accumulates in the nucleus as cells enter S-phase of the cell cycle [29, 34], remains well expressed in mitosis [35, 36], and then undergoes ubiquitination and proteasome-dependent degradation as cells enter G1 phase [36]. Within the S-phase nucleus, BRCA1 is most often directed to different types of foci, which appear as tiny dots by immunofluorescence microscopy (see Figures 1(b) and 2). The BRCA1-positive foci reflect different nuclear complexes and functions of BRCA1, including its role in protecting stalled DNA replication forks [37], regulated silencing of the inactive X chromosome by XIST RNA [38], and other structures relating to epigenetic silencing and chromatin modification [39]. BRCA1 staining has also been detected at the nucleolus [40], although this is not always the case and should be interpreted with caution as the issue of antibody specificity is a recurring problem with sporadic BRCA1 cell staining patterns (discussed in more detail in Subsection 4.3). Immunohistochemical microscopy studies of human breast tumors have frequently detected a shift in BRCA1 from nucleus to cytoplasm in cancers of increasing grade and from patients carrying germ-line *BRCA1* mutations [41–43], and similar observations have been made for breast cancer cell lines expressing mutant forms of BRCA1 [44]. The impact of gene mutations and alternative splicing on BRCA1 nuclear import and distribution is discussed in Section 3. 

### 2.2. The Cytoplasm

BRCA1 shuttles between nucleus and cytoplasm [48]. In the cytoplasm it has been detected at the centrosomes [49, 50] (discussed further in Section 6), where it was found to bind to gamma-tubulin [50]. Subsequent studies employed a combination of cell fractionation, fluorescence microscopy and electron microscopy to identify the phosphorylated form of BRCA1 at mitochondria [51–53] (see also Section 7) and BRCA1 in complex with Bcl-2 at the endoplasmic reticulum [53]. These specific cytoplasmic associations may be focal points for regulation of apoptosis and centrosome-related cell cycle checkpoints that decide mitotic progression. 

## 3. Nuclear Import 

### 3.1. Import Mechanism and Regulation of the Pathway

BRCA1 is actively imported into the cell nucleus by two distinct pathways [16, 17]. One of these involves interaction of the two nuclear localization signals (NLSs) located in the middle of the protein (see Figure 1(a)) [54, 55], with importin-alpha/beta receptors responsible for translocating NLS-cargo through the nuclear pore complex (NPC) into the nucleus (reviewed in [56]). BRCA1 splice variants, in which the NLSs are lost due to splicing out of exon 11, enter the nucleus [30] by accessing a second import pathway that requires the BARD1 binding partner. This alternate mechanism does depend on the importins, but is mediated through a piggy-back mechanism in which BRCA1 binds to BARD1, which then utilizes its own NLSs to chaperone nuclear entry of BRCA1 [57]. More recently, Qin and colleagues [58] postulated that similar binding of the SUMO-dependent E3 ubiquitin ligase, Ubc9, to the N-terminus of BRCA1 could also stimulate its nuclear import and localization. It is therefore possible that fluctuations in different signaling pathways, or in the expression of binding partners, will influence BRCA1 nuclear import, and indeed the targeting of protein import has been linked to disease and pathological conditions such as cancer [59, 60]. In relation to its role in DNA repair, it is thus relevant that nuclear localization of BRCA1 was found to increase transiently within two hours after DNA damage of cells by ionising radiation or etoposide treatment [52], consistent with a coordinated protection against genotoxic stress. It is not yet known if this heightened BRCA1 nuclear expression reflects stimulation of nuclear import or changes in export or nuclear/cytoplasmic retention.

### 3.2. Regulation by Cytoplasmic Retention

Nuclear localization can also be diminished by the enhanced cytoplasmic retention of BRCA1 by specific proteins or structures. The first such retention factor identified was BRAP2 (BRCA1-binding protein 2), a protein that binds to the BRCA1 NLSs and thereby antagonizes BRCA1 association with the nuclear import receptor, importin-alpha [61]. BRAP2 inhibition of NLS-dependent nuclear import was subsequently shown to depend on the phosphorylation of residues that flank the NLS of BRCA1 [62]. BRCA1 is also trapped in the cytoplasm following overexpression of the antiapoptotic factor Bcl-2, which redirects BRCA1 to mitochondria and endoplasmic reticulum [53]. It is not yet clear, however, if these observations are restricted to conditions of Bcl-2 overexpression. In addition, the membrane serine/threonine protein kinase B-alpha (termed AKT1 kinase) was reported to repress homologous recombination mediated DNA repair through the cytoplasmic retention of BRCA1 and Rad51 [63]. This mode of cytoplasmic anchorage was claimed to correlate with the upregulation of AKT1 in sporadic breast cancers, perhaps enhancing levels of genomic instability through partial suppression of DNA repair.

### 3.3. Impact of Cancer Mutations on Nuclear Localization

The two main functionally sensitive regions of BRCA1 targeted by gene mutations are the amino and carboxy terminal ends. There is evidence that substitution/termination mutations within the carboxy-terminal BRCT domain (see Figure 1) (a region common to DNA damage response proteins; see [64, 65]) impede nuclear import of BRCA1. A range of such BRCT mutations were introduced into full-length BRCA1 and found to block nuclear localization [66]. A similar observation was made by Elstrodt et al. [44] in different breast cancer cell lines with BRCT-mutated BRCA1. This issue was previously discussed [16] and thought to reflect gross alterations in the global protein conformation of BRCA1, consistent with known effects of such mutations [67, 68]. Similar testing of the N-terminal C61G RING domain mutation did not reveal any change in nuclear localization by some investigators [57, 66], although a recent study by Qin et al. [58] reported that the C61G mutation elevated cytoplasmic staining of BRCA1, possibly due to reduced binding of the Ubc9 ubiquitin ligase. 

## 4. Nuclear Export 

Hundreds of proteins shuttle in and out of the nucleus by active transport, mediated through interaction with nuclear import and export receptor proteins. The primary nuclear export receptor is CRM1 (chromosome region maintenance protein 1) that binds to protein cargo at the nuclear export signal (NES), a peptide recognition site characterized by large side-chain hydrophobic amino acids spaced according to a loose consensus [69, 70]. The role of CRM1 in protein nuclear export is now recognized as an important regulator of protein function in cancer [70, 71].

### 4.1. BRCA1 Is a Nuclear Shuttling Protein Regulated by BARD1

In 2000, an NES was identified and characterized at the N-terminus of BRCA1, located adjacent to the RING domain [48] (see Figure 1). This sequence (81-QLVEELLKIICAFQLDTGL-99) facilitated CRM1-dependent export of BRCA1 from the nucleus [48]. A second NES, also at the N-terminus (residues 22–30), was later reported by Thompson et al.[72]. Interestingly, the primary NES (81-99) corresponds exactly to one of the two alpha-helices that were later found to flank the RING domain and form the four-helix bundle interaction interface with BARD1 [73]. To investigate the impact of BARD1 binding, detailed mutagenesis and biochemical studies were performed, leading to the conclusion that binding of BARD1 masks the BRCA1 export signal and prevents association with CRM1, thereby causing nuclear retention of BRCA1 [57, 74]. Subsequently, it was revealed that BARD1 itself comprises an NES at the same relative position within the heterodimerization region, and that in fact both proteins are subject to nuclear entrapment upon dimerization [75]. This reciprocal regulatory mechanism of nuclear shuttling is unique and provides an explanation for the almost exclusive detection of BRCA1/BARD1 dimer complexes in the nucleus in both fixed cells [57] and living cells [76].

### 4.2. Regulation of Nuclear Export in Cell Cycle and Apoptosis

BRCA1 undergoes several posttranslational modifications including phosphorylation, ubiquitination and SUMOylation [6, 77], which could influence its nuclear trafficking. In 2003, Okada and colleagues [34] showed that BRCA1 phosphorylation at serine 988 changed during cell cycle progression and that the phosphorylated form redistributes to the perinuclear region of the cell in S-phase after DNA damage treatments. A later study by Glover-Collins and Thompson [78] used immunofluorescence microscopy of synchronised cells to show evidence that BRCA1 cytoplasmic localization increased during the early stage of S-phase, a phenomenon partly linked to CRM1 mediated nuclear export as determined from treatments with the CRM1 inhibitor, leptomycin B. This group suggested that the cell cycle-associated nuclear export of BRCA1 involved a calcium-dependent mechanism, although further verification of this finding and it's implications remain to be explored. 

 The transient overexpression of a BRCA1 cDNA is known to elicit a p53-independent apoptotic response in breast cancer cells [79, 80]. Interestingly, the nuclear export of BRCA1 was directly linked to this proapoptotic activity, which was diminished by mutation of the amino-terminal NES and by BARD1-induced nuclear retention of BRCA1 [74]. Conversely, BARD1-mediated p53-dependent apoptosis was also stimulated by its nuclear export and reduced by coexpression of BRCA1 [75]. It was postulated that the BRCA1/BARD1 heterodimer maintains a cell survival function when localized to the nucleus, due partly to its role in DNA repair, but that BRCA1 and BARD1 individually elicit apoptosis correlating with their independent export to the cytoplasm [11, 16, 74, 75]. A later study by others also claimed to observe a correlation between cytoplasmic localized BRCA1 and activation of the intrinsic caspase cleavage pathway, in particular after DNA damage [81, 82]. The actual mechanism by which cytoplasmic-localized BRCA1 elicits cell death is not fully understood and is likely to reflect multiple factors including a shift in the balance between BRCA1 and BARD1 (either protein in the undimerized form has enhanced shuttling and apoptotic potential), potential triggering of a centrosomal checkpoint [83], or direct action at mitochondria [51, 84].

### 4.3. A Potential Role for BRCA1 Nuclear Export in DNA Repair?

One group reported that a fraction of endogenous BRCA1 was exported from nucleus to cytoplasm within one hour of ionizing radiation-induced DNA damage in MCF-7 breast cancer cells [82, 85]. It was claimed that DNA damage induced a p53-dependent nuclear export of BRCA1, however, the results have been questioned by Brodieand Henderson[52] who showed that the antibody used by Feng et al. [85] cross-reacts with other cytoplasmic proteins under the fixation conditions used, and that a more extensive biochemical testing and cell staining actually revealed no evidence that BRCA1 nuclear export is affected by DNA damage [52]. In fact, careful analysis of cell fractions shows the exact opposite situation with nuclear BRCA1 increasing early after ionising radiation, consistent with its role in DNA repair [52]. The issue of antibody cross-reactivity, combined with poorly quantified or inconsistent subcellular fractionation and cell imaging experiments, has hampered the integrity of BRCA1 investigations on and off over the years and emphasizes the need for all investigators in future to validate their findings, for instance, by proving that any staining pattern observed with BRCA1 antibodies is eliminated by siRNA knockdown of BRCA1. Moreover, and of particular relevance to some recent studies (e.g., [82]), the detection of cytoplasmic staining of BRCA1 should not automatically be interpreted as resulting from nuclear export, as it can also arise from changes in the rates of translation, or cytoplasmic or nuclear turnover, or from retention at specific cytoplasmic structures, as facilitated by BRAP2 [61] or loss of Ubc9 SUMOylation activity (see [58]). Currently, the main validated function of BRCA1 nuclear export appears to be linked to its role in centrosome duplication, as indicated directly by testing a range of different NES- and NLS-mutated forms of BRCA1 [46] (discussed in Section 6).

## 5. Localization and Dynamics of BRCA1 at DNA Repair-Associated Nuclear Foci

The cellular genome is regularly exposed to external stresses and stimuli that damage DNA, causing >200,000 damage events per day. DNA breaks can be induced by ionizing or ultraviolet radiation, carcinogens, oxidative chemicals, and free radicals, in addition to clinically administered chemotherapeutic agents. Repair of damaged DNA is vital for cell proliferation and survival [86]. The cell responds to DNA damage, depending on the type of DNA lesion induced, either by stalling cell division and repairing the damaged DNA, or by triggering a cell death (apoptotic) response. BRCA1 is implicated in several repair pathways, in particular homologous recombination repair of double-strand DNA breakages.

### 5.1. Targeting of BRCA1 to Nuclear Foci

Endogenous BRCA1 appears in the nucleus at discrete “foci” during DNA replication [35, 87, 88]. These foci appear as microscopic spots in the nucleus when visualised by microscopy and correlate with the role of BRCA1 as a regulator and protector of stalled DNA replication forks [37] (Figure 2(c)). The best-known function of BRCA1 is in DNA repair, and in response to induction of single-strand or double-strand DNA breaks BRCA1 forms different types of protein complexes at foci to repair the damage. The targeting of BRCA1 to XRCC1-positive DNA repair complexes, in response to alkylating agent-induced single-strand DNA breaks, involves a combination of the BRCA1 amino terminus (aa 1–304) and the central SQ/TQ cluster domain region (1078–1312) which binds to DNA damage response kinases such as ATR [89]. On the other hand, cooperation between the amino terminus (1–304) and the carboxy-terminal BRCT domains (1620–1863) is required to mediate efficient targeting of BRCA1 to double-strand DNA breaks caused by ionising radiation [45] (Figure 2). Thus, distinct sequence elements are responsible for directing BRCA1 to different types of DNA repair complex.

 The BRCA1-associated DNA repair complexes that form after ionising radiation have been studied in detail biochemically, most often using the carboxy-terminal BRCT domains of BRCA1 to screen for protein partners. This general approach has identified several types of chromatin repair and modification complexes. These are the BRCA1-A (controls G2-M checkpoint and DNA repair), BRCA1-B (DNA replication checkpoint), BRCA1-C (DNA resection and G2-M checkpoint), and BRCC (homologous recombination mediated DNA repair) complexes (reviewed recently in [6, 22, 77, 90, 91]). Most studies use ionising radiation (IR) to elicit consistent DNA double-strand breaks and typically detect the recruitment of BRCA1 into 10–100 distinct nuclear foci per cell within 1 hour, as illustrated in Figure 2 (reviewed in [77]). IR induces a G2-M cell cycle arrest that can be rescued by BRCA1 through activation of Chk1 [83].

**Figure 3:**
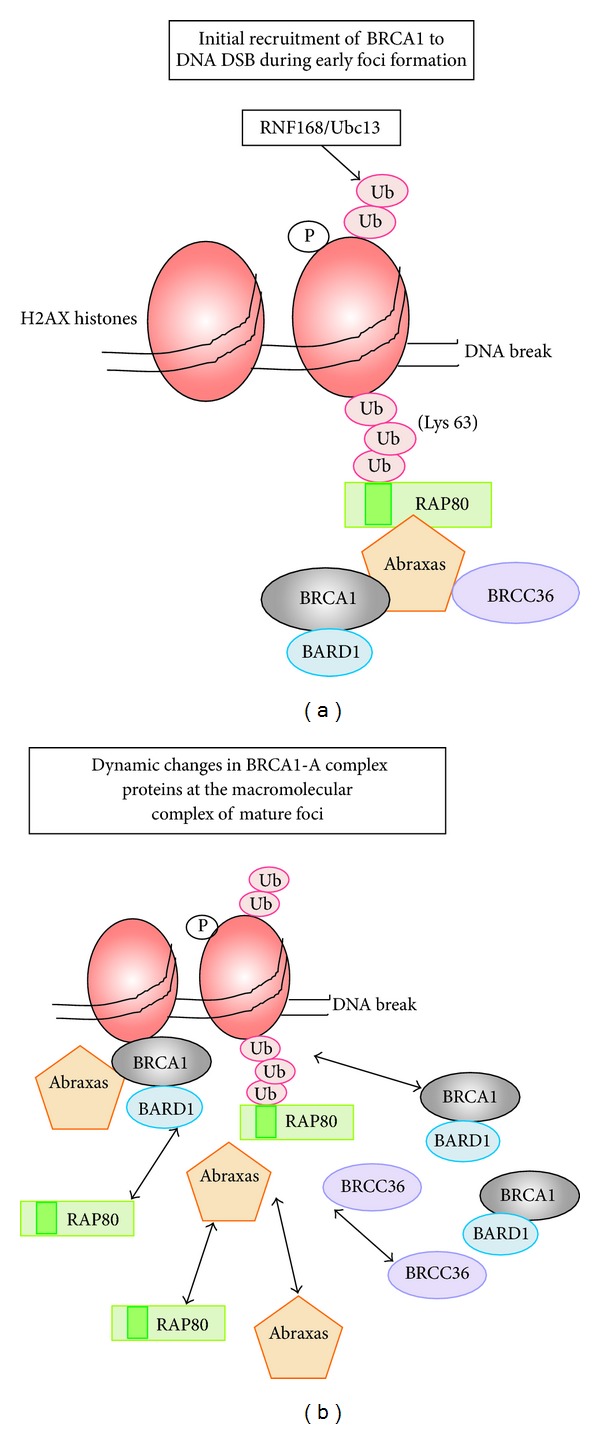
*Models for the hierarchical assembly and subsequent dynamics of the BRCA1-A DNA repair complex at foci.* (a) A summary of recent understanding of the initial stages of BRCA1 recruitment to DNA damage foci (i.e., complexes formed at DNA double-strand breaks induced by IR). Upstream events include roles of ATM, MDC1, and RNF8 leading to RNF168/Ubc13-mediated ubiquitination of chromatin break-point components such as phospho-H2AX histones. This tagging of DNA breaks through the accumulation of Lys-63 ubiquitin (Ub, red) moieties leads to recruitment of RAP80 (green) via its ubiquitin-binding domain (dark green). RAP80 then recruits Abraxas/CCDC98 (orange) which in turn binds BRCA1 (grey), BARD1 (blue), BRCC36 (violet), and some additional components. This diagram exemplifies the “static” model for BRCA1 recruitment to foci shown in many previous review articles. (b) A new model for BRCA1-positive foci emphasizes the fact that most components undergo changes in their internal protein-protein interactions as the focal complex develops, such that once formed even the upstream RAP80 protein, previously thought to anchor the complex, is no longer required for BRCA1/BARD1 foci targeting (discussed in Section 5). In fact, at such mature foci, most components exchange rapidly at the focal complex, with RAP80 being one of the most dynamic components. The dynamic organisation of the BRCA1-A complex at foci has been proposed to comprise a small (~20% of total protein pools) immobilized fraction of each protein that forms an open scaffold, at which the dominant and dynamic pool (80%) transiently associates through rapid exchange [76].

 IR-induced DNA damage leads to the activation of PI-3 kinases ATM and ATR, which phosphorylate BRCA1 and other DNA repair proteins at IR-induced foci. A cascade of protein modifications and interactions result in BRCA1 accumulation at these foci. Phosphorylated histone H2AX (gamma-H2AX) is the first to arrive at double-strand breaks [92] (Figure 3(a)), initiating a hierarchical cascade of protein modifications and interactions that lead to recruitment of BRCA1 to the IR-induced repair foci at distinct DNA repair protein complexes. The so-called BRCA1-A complex comprises RAP80, Abraxas/CCDC98, the deubiquitinase BRCC36, BRCC45 and MERIT40, which are thought to target BRCA1 to IR-induced foci through the interaction of RAP80 with polyubiquitin chains such as is thought to occur at histone gamma-H2AX [6, 22] (see Figure 3(a)). The accumulation of lysine 63-linked ubiquitin conjugates at H2AX is indeed required for foci formation and is regulated by dynamic action of specific ubiquitin ligases including RNF168 and RNF8 [93].

### 5.2. Implications of the Dynamic Organisation of BRCA1 Complexes at DNA Repair Foci

**Figure 4:**
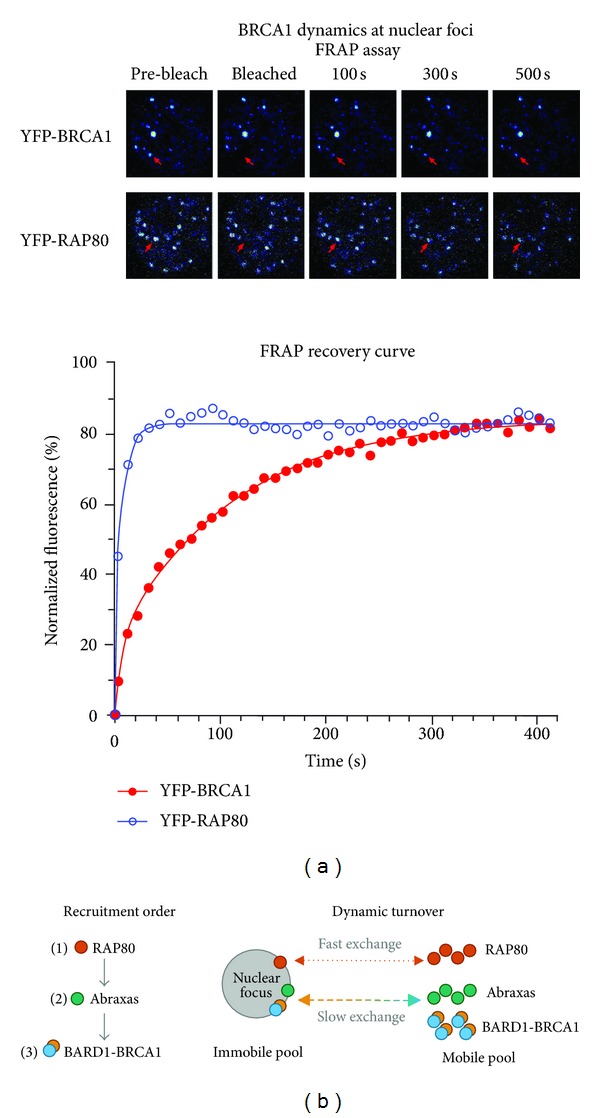
*Dynamic exchange of BRCA1 at nuclear foci determined by photobleaching assays.* Several teams have studied the kinetics of recovery of DNA repair factors at DNA breaks by fluorescence recovery after photobleaching (FRAP) assay, although in the case of BRCA1-A complex proteins this was most often at laser-induced microirradiation lines that span the entire nucleus [77]. (a) A recent study used stable inducible cell lines to quantify and compare the dynamics of BRCA1 and associated DNA repair proteins at individual IR-induced foci in the nucleus [76]. This revealed a rapid recovery of different components, including BRCA1 and BARD1 which moved to and from foci as a dimer, and an extremely rapid on-off rate of the upstream factor RAP80. This is illustrated by rapid recovery of YFP-RAP80 at an individual focus (red arrow) following laser bleaching of the focal fluorescence (see also recovery curve graph). (b) The findings of that work are consistent with a hierarchy of protein targeting to foci (see left panel), but present a new perspective in terms of the dynamic nature of components at repair foci (see right panel). This figure was adapted from [76].

When studied *in vitro* in isolation, both the amino-terminal RING domain and carboxy-terminal BRCT domains of BRCA1 can multimerize independently [94, 95], sometimes at DNA break points [94]. It is of relevance that the same sequences are required for optimal targeting of BRCA1 to IR-induced foci to assemble large macromolecule protein complexes [45]. In the context of the BRCA1-A DNA repair complex, most models illustrating this huge assemblage show static protein interactions based on data from biochemical assays, rather than the likely dynamic nature of these complexes (see Figure 3). For example, repair foci have an average expected diameter of ~100 nm and comprise large macromolecular assemblies of thousands of proteins, some of which might be fixed at chromatin while others are exchanging to and from the complex with rapid dynamics in the range of seconds [76, 96]. Moreover, the mobility or anchorage of proteins at foci will change upon posttranslational modification by phosphorylation, ubiquitination and SUMOylation [96], which has indeed been implicated for BRCA1 localization at foci (see [77]). The upstream regulator RAP80 is required to initially target BRCA1/BARD1 and associated proteins to DNA double-strand breaks; however, recent findings indicate that RAP80 does not remain as a fixed anchor of the BRCA1-A complex at foci, as generally presumed in the literature, but its interactions with BRCA1 and associated factors are transient and change after the complex forms (Figure 3(b)). Indeed, a detailed analysis of individual IR-induced foci in stable-inducible MCF-7 cells revealed that the majority (80%) of YFP-tagged RAP80 at mature foci is extremely dynamic [76] (Figure 4(a)). In fact, while YFP-tagged BRCA1/BARD1 and Abraxas/CCDC98 were also dynamic, RAP80 was under the most dynamic flux at foci (Figure 4(a)), showing that it is does not function to anchor the complex once foci start to form. When MCF-7 breast cancer cells are subjected to detergent extraction to remove weakly bound proteins, microscopic imaging revealed that from as little as 30 min after irradiation a small immobile pool of BRCA1 remains detectable at foci even after RAP80 is completely removed, and the BRCA1 staining increases with time [76]. It is likely that changes in internal protein-protein interactions occur very rapidly after the BRCA1-A complex components begin to accumulate at the chromatin break points (Figures 3 and 4(b)). It is not yet known what anchors the immobile pools (~20% of total protein at foci) of BRCA1/BARD1, Abraxas, or MERIT40 at nuclear foci; however, it is likely to be associated with chromatin or possibly with the nuclear matrix [97]. 

 The distribution of BRCA1 at nuclear foci is uniform as determined by electron microscopy, and consistent with an immobile open scaffold of ~100 BRCA1 molecules per DNA break, with transient contact made by another ~500 BRCA1 molecules that dynamically exchange with the surrounding nucleoplasm [76]. Similar high copy numbers of BARD1 and Abraxas molecules were determined at individual foci, although RAP80 was even more abundant and displayed twice as many molecules (~1200 copies) per focus [76]. The abundance and ultra-fast dynamic exchange of RAP80 at repair foci may be important for signaling a rapid response to external stimuli, empowering it with the ability to “tune” the function of BRCA1/BARD1 in homologous recombination mediated DNA repair [98]. An additional consequence of having such large mobile pools is that the bulk of BRCA1 and its partners could quickly be dispatched, within 10–20 seconds, from a focal complex once the DNA repair event is complete. Such termination and disassembly of foci can be controled by ubiquitin-dependent proteasome degradation of individual components [99], and this would prove very efficient if coupled with altered protein dynamics or release from the immediate stable chromatin-protein complex.

## 6. Localization and Function at the Centrosome

Centrosomes are nonmembranous organelles located in the cellular cytoplasm and duplicate with each cell division. The primary function of centrosomes is to nucleate assembly of the microtubule network during interphase, and to prime assembly of spindle microtubules that form the mitotic spindle for chromosome segregation [100]. Genetic mutations, or altered expression, of BRCA1 correlate with alterations in centrosome number and integrity of the microtubule cytoskeleton observed in breast cancers [101–103]. Recent progress has been made in determining how BRCA1 is recruited to the centrosome and its regulated dynamics and functional roles.

### 6.1. Protein Sequences That Target BRCA1 to the Centrosome

**Figure 5:**
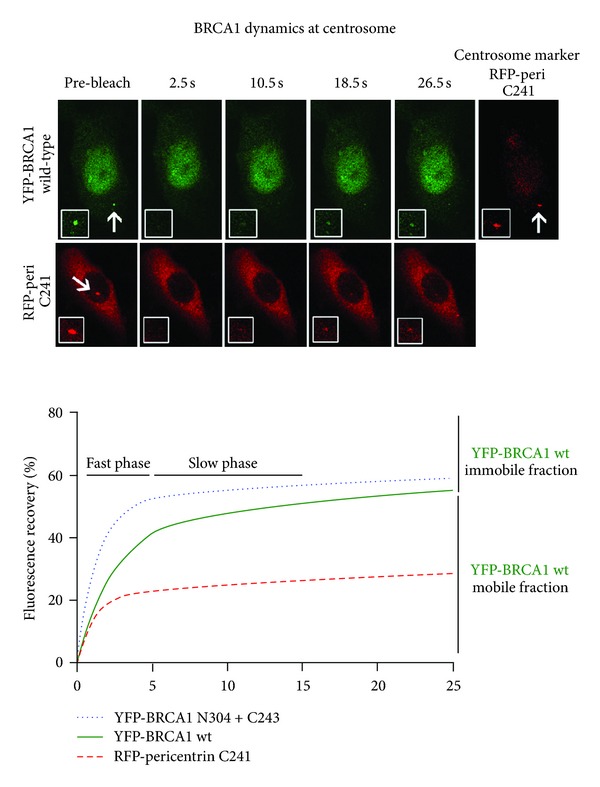
*BRCA1 exists in dynamic and immobile states at the centrosome.* The mobility of BRCA1 has been examined at the centrosome by FRAP assay. As shown (top panel), images of transiently expressed YFP-BRCA1 in MCF-7 cells reveal a rapid recovery of BRCA1 fluorescence within 20 seconds after photobleaching at the centrosome (adapted from Brodie and Henderson [46]). In contrast, bleaching of the carboxy-terminal fragment of centrosomal pericentrin (tagged with RFP) recovered to a lesser extent. Quantification of fluorescence recovery is shown in the graph (lower panel), revealing that ~40% of wild-type YFP-BRCA1 is immobile at the centrosome, whereas ~60% is under dynamic exchange. Note that an internal deleted form of BRCA1 (N304 + C243), that lacks the two recently described gamma-tubulin binding sites [104], displays a moderately faster rate of recovery after bleaching, suggesting that interaction with gamma-tubulin contributes to some of the transient interactions between BRCA1 and centrosome. These data were originally published in Brodie and Henderson [46], ©  the American Society for Biochemistry and Molecular Biology, and reproduced with permission.

BRCA1 was first detected at the centrosome by Hsu and colleagues, who further showed that BRCA1 bound to the centrosomal component gamma-tubulin via an internal BRCA1 sequence comprising amino acids 510–622 [49, 50]. Tarapore et al. [104] recently identified a second gamma-tubulin binding domain in BRCA1 (residues 802–1002), which was proposed to contribute to BRCA1 localization at the mother centriole, and later at the daughter centriole prior to centrosome duplication, as determined by scoring of detergent-permeabilized cells. Under these experimental conditions, only a strongly attached pool of BRCA1 would be detectable at the centrosome, suggesting that gamma-tubulin anchors a subpool of BRCA1. A more exhaustive deletion mapping study has revealed that a combination of the N- and C-terminal sequences of BRCA1 (amino acids 1–304 + 1620–1863, see Figure 2(a)) were sufficient to target BRCA1 to the centrosome in both fixed cells and live cells [46]. Interestingly, when Brodie and colleagues used live cell photobleaching assays (FRAP; fluorescence recovery after photobleaching) to analyse a YFP-tagged form of BRCA1 lacking both internal gamma-tubulin sequences, this deleted form displayed moderately faster recovery dynamics indicative of reduced retention at the centrosome (see Figure 5). These collective findings imply that multiple sequences may contribute to the overall targeting, exchange, and retention of BRCA1 at the centrosome or its subcompartments. 

### 6.2. BRCA1 Regulates Microtubule Nucleation/Elongation and Centrosome Duplication

BRCA1/BARD1 functions as an E3 ubiquitin ligase, and this enzymatic activity was previously implicated in the monoubiquitination of *γ*-tubulin at lysines 48 and 344, leading to a reduction of gamma-tubulin-primed microtubule aster formation (nucleation of microtubules) [105] and the inhibition of centrosome duplication [106, 107]. Therefore, the ability of BRCA1 to bind and ubiquitinate gamma-tubulin suppresses inappropriate centrosomal function typically observed in some breast cancers. The recent analyses of BRCA1 and BARD1 in living cells revealed that both proteins traffic very efficiently to the centrosome on their own [46, 108], thus it is possible that the BRCA1/BARD1 dimer assembles after correct placement of BRCA1 within the pericentriolar matrix region where the gamma-tubulin ring complex forms. Analysis of overexpressed sub-fragments of BRCA1 indicated that BRCA1 repression of microtubule nucleation is not strictly dependent on Ub ligase activity, as expression of the nonubiquitinating gamma-tubulin binding sequence (802–1002) was sufficient to impede aster formation [104]. BRCA1 has also been reported to ubiquitinate a different centrosomal protein, nucleophosmin (NPM1) [109]; however, the functional consequences have not yet been determined.

### 6.3. Regulation of Centrosome Targeting by CRM1, Phosphorylation, and BARD1

**Figure 6:**
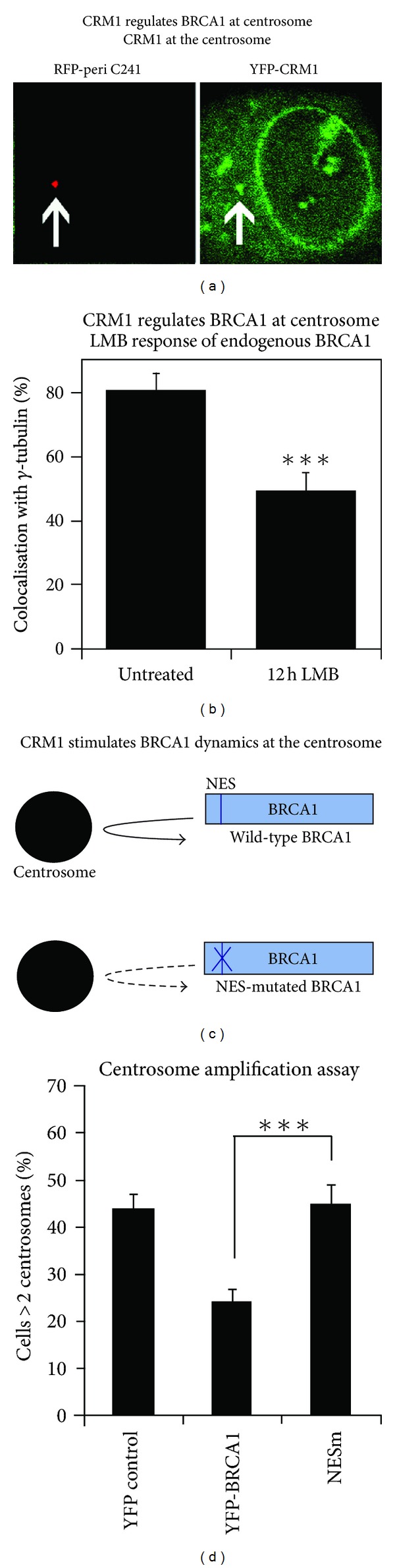
*BRCA1 nuclear export stimulates targeting to the centrosome.* BRCA1 is known to be exported from nucleus to cytoplasm by the CRM1 export receptor [48, 72]. (a) CRM1 has been implicated in centrosome function and when overexpressed as a YFP fusion is detectable at the centrosome by microscopy (CRM1 shown in green, centrosome marker pericentrin shown in red). (b) The inhibition of CRM1 activity by treatment with leptomycin B (LMB) results in reduced staining of endogenous BRCA1 at the centrosome. (c) A range of recent FRAP experiments [46] showed that mutation of the BRCA1 NES caused an increase in BRCA1 exchange rate at the centrosome, consistent with reduced retention. (d) Moreover, mutation of the NES abolished the ability of ectopic wild-type BRCA1 to control rampant centrosome amplification after exposure of HCC1937 breast cancer cells to ionising radiation. This implies that CRM1 contributes to the role of BRCA1 in tight regulation of centrosome duplication. These data were originally published in Brodie and Henderson [46], ©  the American Society for Biochemistry and Molecular Biology, and reproduced with permission.

The first live cell FRAP assays of BRCA1 at the centrosome revealed two main centrosomal pools: one of rapid mobility and exchange (60%), and a very slow exchanging pool (40%) that appeared immobile during the 30 s time period studied [46] (Figure 5). Mutation of the NES, or treatment of cells with leptomycin B, a CRM1 export inhibitor, caused a reduction in BRCA1 transport to the centrosome and in its overall rate of exchange and retention [46] (Figures 6(a) and 6(c)). This contrasts with the more dramatic effect of leptomycin B reported for nucleophosmin, wherein a block to nuclear export supposedly caused dissociation of nucleophosmin from the centrosome [110], correlating with centrosome amplification. Interestingly, CRM1 was not essential for BRCA1 localization but did stimulate its targeting and movement to the centrosome [46]. Moreover, mutation of the NES abolished the ability of full-length BRCA1 to regulate centrosome amplification in DNA damaged BRCA1-mutant breast cancer cells (illustrated in Figure 6(d)). It is of interest to note that in addition to BRCA1 and nucleophosmin, BRCA2 nuclear export was also implicated in regulating centrosome duplication [111].

 The recruitment of BRCA1 to the centrosome does not depend on BARD1, in fact the BRCA1/BARD1 dimer displayed a faster dynamic turnover, and was less well retained than monomeric BRCA1 [46]. This suggests that the proteins might move to the centrosome separately and assemble as a dimer only transiently, perhaps long enough to ubiquitinate substrates such as gamma-tubulin before dissociating from the structure. There is further evidence that binding to BRCA1, and phosphorylation of BRCA1 at serine 308, by Aurora A kinase contributes to centrosome retention of BRCA1 in live cells and also to its role in inhibition of centrosome duplication [46]. These observations correlate with the role of Aurora A kinase in inhibiting microtubule nucleation through perturbation of BRCA1 ubiquitin ligase activity [106]. In future experiments it will be important to define more precisely whether CRM1 directs BRCA1 to a specific subcompartment of the centrosome (e.g., mother or daughter centriole compared to pericentriolar matrix), and how different binding partners influence the exchange and functionality of BRCA1 at these sites.

### 6.4. Regulation of Mitotic Spindle and Epithelial Cell Polarity

As cells progress through the cell cycle, the duplicated centrosomes mature in late G2 phase to form the mitotic spindle pole body responsible for assembling the mitotic spindle. BRCA1 has long been implicated in the mitotic spindle checkpoint, a cellular safeguard that maintains DNA integrity by stopping cells with misaligned chromosomes from exiting mitosis [112]. Studies in Xenopus laevis oocytes by Joukov and colleagues [113] revealed that BRCA1/BARD1 ubiquitin ligase activity is required for assembly of the mitotic spindle pole and that this involves binding of BRCA1 to the spindle pole proteins TPX2, NuMa, and XRhamm. In human cells, BRCA1 binds to RHAMM (receptor for hyaluronan-mediated mobility), which is then ubiquitinated by BRCA1/BARD1 [114]. The interplay between BRCA1 and RHAMM is linked to breast cancer, both through *BRCA1* gene mutations in familial breast cancer and low-penetrance genetic variation at the HMMR locus (encoding RHAMM) associated with sporadic breast cancer [115]. The interlinked network of BRCA1/BARD1, RHAMM, Aurora A, and TPX2 is important for efficient apicobasal polarity of epithelial cells, and disruption of this through altered microtubule dynamics or altered mitotic spindle can increase the risk of cancer [115].

## 7. Localization at Mitochondria and Implications for Apoptosis and Drug Sensitivity

BRCA1 has been detected at cellular mitochondria by immunofluorescence microscopy and electron microscopy [51], suggesting that BRCA1 is targeted to the inner mitochondrial matrix where it might play a role in regulating mitochondrial DNA repair. BRCA1 becomes hyperphosphorylated during S-phase, and it is this form which locates at mitochondria [51, 52]. BARD1 has also been detected at mitochondria [84]. When the two proteins were compared for mitochondrial distribution relative to the total cytosolic pool, it was found that BARD1 was preferentially localized at the mitochondria with a ratio ~4-fold higher than that of BRCA1 [52]. The targeting of phospho-BRCA1 and BRCA1/BARD1 complexes to mitochondria could serve to repair damaged mitochondria genes as part of its protective cellular role. In this regard, it is also notable that cytoplasmic BRCA1 was found to suppress macroautophagy by reducing formation of autophagic vacuoles in breast cancer cells [116]. In contrast, changes in BRCA1 expression or mutation status could increase apoptotic responses under specific conditions. For instance, BRCA1 is implicated in UV-induced caspase-3 activation [117], and in the induction of caspase-9 cleavage in response to ionising radiation [81]. Some of these responses are altered in breast cancer cells expressing different BRCA1 mutations, in line with previous predictions that mutated forms of BRCA1 will determine tumor cell sensitivity or resistance to different types of chemo- or radiotherapies [7]. The role of mitochondrial-localized BRCA1 in anticancer drug responses is a clinically relevant area in need of further investigation.

## 8. Conclusions and Future Directions

Since the initial discovery of BRCA1 and its role in the cellular DNA damage response, the last few years have witnessed a broad expansion in the number of interacting protein partners, the cellular localization sites, and the functions of this critical breast cancer regulatory protein. This paper has focused on the cellular location and transport of BRCA1 and how these pathways regulate different types of BRCA1 activity. Recent discoveries as described above on the dynamic nature of BRCA1 trafficking now open up new areas to explore, including use of proteomics approaches to better define the composition of BRCA1 protein complexes at specific subcompartments of the nucleus, centrosome and mitochondria. A specific mass spectrometry screening for BRCA1 partners has not yet been systematically performed using purified subcellular fractions and will likely yield important new binding partners that could regulate or mediate BRCA1 activity. Furthermore, it will prove interesting to evaluate how different partners and modifications (e.g., SUMOylation) of BRCA1 affect its retention, mobility, and activity at these cellular sites. In relation to cancer, continued investigation into how specific gene mutations alter BRCA1 cell cycle checkpoint functions through the modulation of its transport and/or activity could provide new insights, and the study of intracellular shuttling of BRCA1 and its misregulation by mutations might have additional implications for clinical applications of variability in tumor cell sensitivity to chemotherapy.

## Abbreviations

ATR: Ataxia telangiectasia and Rad3-related protein
BARD1: BRCA1-associated RING domain protein 1
BRCA1: Breast cancer susceptibility gene 1
CRM1: Chromosome region maintenance protein 1
IR: Ionising radiation
NLS: Nuclear localisation signal
NES: Nuclear export signal
RFP: Red fluorescent protein
YFP: Yellow fluorescent protein.
